# Zinc Methionine Supplementation Impacts Gene and Protein Expression in Calf-Fed Holstein Steers with Minimal Impact on Feedlot Performance

**DOI:** 10.1007/s12011-015-0521-2

**Published:** 2015-10-07

**Authors:** J. E. Hergenreder, J. F. Legako, T. T. N. Dinh, K. S. Spivey, J. O. Baggerman, P. R. Broadway, J. L. Beckett, M. E. Branine, B. J. Johnson

**Affiliations:** Department of Animal and Food Sciences, Texas Tech University, Box 42141, Lubbock, TX 79409-2141 USA; Beckett Consulting Services, Fallbrook, CA 92028 USA; Zinpro, Eden Prairie, MN 55344 USA; Department of Nutrition, Dietetics, and Food Sciences, Utah State University, Logan, UT 84322 USA; Department of Animal and Dairy Sciences, Mississippi State University, Starkville, MS 39762 USA

**Keywords:** β-Adrenergic receptor, Myosin heavy chain, Zilpaterol hydrochloride, Zinc methionine

## Abstract

Providing cattle a more bioavailable zinc (Zn) source prior to administering a beta adrenergic agonist (βAA) may enhance the metabolic pool of primary nutrients that will influence the magnitude of the βAA response. Calf-fed Holstein steers were supplemented with a Zn methionine supplement (ZnMet; ZINPRO^®^; Zinpro Corporation, Eden Prairie, MN) for 115 ± 5 days prior to harvest along with zilpaterol hydrochloride (ZH; Zilmax^®^; Merck Animal Health, Summit, NJ) for the last 20 days with a 3-day withdrawal to evaluate the effects on growth and carcass performance together with gene and protein expression of skeletal muscle, adipose tissue, and fatty acid composition of polar and neutral lipid depots. Steers (*n* = 1296; initial weight = 468.5 ± 0.5 kg) were sorted by weight, blocked by harvest date, and randomly assigned to pens (*n* = 12) and treatments: control (90 ppm Zn from ZnSO_4_) and ZnMet (Control plus 720 mg Zn from ZnMet/hd/d). There were no differences (*P* > 0.05) in growth performance or carcass characteristics. The ZnMet-fed cattle had reduced (*P* < 0.05) abundance of myosin heavy chain (MHC)-IIX, β1-adrenergic receptor (βAR), peroxisome proliferator-activated receptor gamma, and stearoyl-CoA desaturase mRNA in skeletal muscle tissue. The ZnMet cattle had greater (*P* < 0.05) abundance of MHC-II protein, increased MHC-IIA and IIX cross-sectional areas (*P* < 0.05), an increased percentage of MHC-I fibers (*P* < 0.05), and a decreased percentage of MHC-IIX fibers (*P* < 0.05). The combination of ZnMet and ZH had positive biological effects on musculoskeletal tissue; however, these molecular effects were not significant enough to impact overall feedlot and carcass performance.

## Introduction

Organic complexes of Zn, such as Zn methionine (ZnMet) have been shown to possess greater bioavailability than inorganic Zn forms such as Zn oxide (ZnO) or Zn sulfate (ZnSO_4_) [23]. Spears [24] reported that ZnO and ZnMet are absorbed to a similar extent but are metabolized differently following absorption. Lambs supplemented with ZnMet had greater retention of Zn and nitrogen compared to lambs supplemented with ZnO [24]. Zinc methionine has been reported to increase growth and feed efficiency in ruminant animals [25]. Furthermore, ZnMet has reportedly improved carcass quality, increasing the percentage of cattle that grade USDA choice [3, 6, 21].

Zilpaterol hydrochloride (ZH) is a beta adrenergic agonist (βAA), approved for use in cattle under the trade name of Zilmax^®^ (Merck Animal Health, Summit, NJ), and is fed at a rate of 6.8 g/ton (100 % DM) for the last 20 to 40 days of the finishing phase with a 3-day withdrawal prior to slaughter [19]. Zilpaterol HCl has been documented to improve feed to gain (F:G), hot carcass weight (HCW), and dressing percentage when administered orally to cattle [26, 29].

Zilpaterol HCl primarily binds with the beta 2 adrenergic receptor (β2AR), which is the most predominant beta adrenergic receptor (βAR) found in cattle muscle and adipose tissue [12, 13]. Via a secondary messenger signal cascade event, cyclic adenosine monophosphate (cAMP) is activated thereby resulting in protein accretion and lipid catabolism [12, 13]. The β2AR potentially has multiple allosteric binding sites for Zn [12, 27]. It has been suggested that there are two main binding sites for Zn on the βAR [28]; one affects the agonist’s ability to bind to the receptor, while the other affects the antagonist’s ability to bind to the receptor thus increasing cAMP production.

Therefore, providing feedlot cattle with a greater amount of ZnMet prior to administering ZH may provide a greater metabolic pool of primary nutrients that will elicit a greater biological response from the ZH, especially in skeletal muscle tissue. The objective of this study was to determine the effect of supplementing ZnMet (ZINPRO^®^; Zinpro Corporation, Eden Prairie, MN) and ZH on feedlot growth performance, carcass characteristics, skeletal muscle and adipose tissue gene expression, protein synthesis, and fatty acid composition of calf-fed Holstein steers.

## Materials and Methods

The Institutional Animal Care and Use Committee’s approval was not needed for this study, as animals were fed offsite from Texas Tech University at a commercial beef cattle feed yard and harvested at a USDA inspected commercial abattoir [1].

### Animals and Management

Holstein steers (*n* = 1296; 481.5 ± 0.5 kg) were sourced in southern California and fed in a commercial feed yard prior to study initiation. Initial processing of steers occurred upon arrival, and cattle were subjected to vaccination and management practices in accordance with the feedlot protocols. Initial processing procedures were typical for Holsteins fed in the Southwest USA.

Finishing rations were formulated to meet or exceed the National Research Council [14] requirements for growing and finishing beef cattle. Ingredient composition of the finisher diets is shown in Table 1. Treatment diets were fed ad libitum throughout the study.

**Table 1:** Ingredient composition (DM basis) of the experimental finishing diets

Ingredient (%)	Diet 1^a^	Diet 2^a^
Sudan hay	7.54	7.66
Bakery waste	8.17	8.30
Steam flaked corn	–	57.24
Flaked wheat	60.72	–
Wheat straw	2.45	2.49
Liquid urea	–	0.64
Dried distillers grain	9.91	13.43
Fat, yellow grease	5.88	4.78
Supplement	5.33	5.46

Diets were formulated to meet or exceed NRC (1996) requirements for growing–finishing beef cattle

Diets contained 6.8 g/t zilpaterol hydrochloride (Zilmax: Merck, Summit, NJ) when fed for the final 20 days of the finishing period

^a^Diet 1 was fed for 174 days, and Diet 2 was fed for 38 days

Complete ration composition profiles were obtained throughout the study (Table 2). Individual ration samples were sent to Michigan State University (Lancing, MI) for analyses. Ration samples were analyzed for moisture, crude protein (CP), acid detergent fiber (ADF), calcium (Ca), phosphorus (P), potassium (K), and Zn (Table 2). Pens were observed daily by trained personnel to identify and remove steers with observable signs/symptoms of health and/or lameness issues.

**Table 2:** Calculated nutrient and laboratory analysis of diet samples

	Diet designation		
Nutrient	Formulated	CON Finishing^a^	ZnMet Finishing^b^
Dry matter (%)	83.5	86.60	85.40
Crude protein (%)	13.3	15.80	15.70
Acid detergent fiber (%)	–	10.00	10.90
Neutral detergent fiber, (%)	–	19.00	20.80
Calcium (%)	0.74	0.96	0.85
Phosphorus (%)	0.41	0.45	0.43
Magnesium (%)	0.25	0.25	0.25
Potassium (%)	–	1.02	1.00
Sodium (%)	–	0.28	0.29
Sulfur (%)	0.24	0.27	0.26
Iron (ppm)	–	317	352
Zinc (ppm)	94/160^c^	122	163
Copper (ppm)	23	28	28
Manganese (ppm)	61	77	72
Molybdenum (ppm)	-	1.00	1.20
Cobalt (ppm)	0.70	1.40	1.30
Selenium (ppm)	0.40	0.60	0.59
Iodine (ppm)	0.80	1.10	1.14

Samples analyzed by Forage Testing Laboratory, Ithaca, NY, except for Se and I which were analyzed by Michigan State University, Diagnostic Center for Population and Animal Health

^a^CON: Control 90 ppm Zn added from ZnSO_4_

^b^ZnMet: Control plus 720 mg Zn added from ZINPRO^®^ zinc methionine

^c^Formulated Zn levels for CON and ZnMet diets, respectively

### Experimental Design and Treatments

Steers were randomized to pens by receiving lot at the time of terminal steroidal implant administration. Each lot was sorted by body weight (BW) to form two pens (one pen/treatment) and blocked by harvest date. A total of 12 pens (*n* = 108 head/pen) were utilized and served as experimental unit. Pens (*n* = 12) were randomly assigned to one of two treatments: 1) 90 ppm Zn from ZnSO_4_ (CON; *n* = 6 pens) or CON plus 720 mg/steer/day of Zn from ZINPRO (ZnMet; *n* = 6 pens). Treatment diets were administered on the final 115 ± 5 days of the finishing period. Zilpaterol HCl was fed for the final 20 days with a 3-day withdrawal prior to shipping and slaughter. Upon trial initiation, steers were weighed individually (initial BW was reduced by 4 % to represent a standard industry shrink).

### Harvest and Carcass Evaluation

Prior to shipment to the abattoir, steers were weighed by pen on a platform scale (final BW was reduced by 4 % to represent a standard industry shrink). Steers were transported 35 km to a nearby commercial abattoir and subsequently harvested under USDA-FSIS inspection. Pens of cattle were maintained as lots when presented for harvest.

Carcasses were chilled approximately 36 h prior to grading. Individual carcass measurements included HCW, dressing percentage (DP), loin muscle area (LMA), and marbling score (MS) and were determined via a digital camera grading system. Yield grade (YG) and quality grade (QG) information was recorded as assigned by USDA graders. Dressing percentage for each pen was calculated as the mean HCW / mean shrunk (4 % pencil shrink) final live weight × 100.

### Collection of Skeletal Muscle and Adipose Tissue

Steers (*n* = 40; 20 steers/treatment) were randomly selected for biochemical analysis of muscle and adipose tissue. Muscle and adipose tissue were collected from the *semimembranosus* muscle of carcasses within 45 min of harvest. The pre-rigor *semimembranosus* tissue sample was cut into thirds. For immunohistochemical analysis, one cut of the samples was placed in a clear frozen section compound (VWR International, Randor, PA), frozen using dry ice-chilled 2-methyl-butane and then placed in a cooler of dry ice. The other two cut samples were placed into a whirl-pack for RNA, protein analysis, and fatty acid analysis, flash frozen in liquid nitrogen, and placed in a cooler of dry ice. The adipose tissue was cut in half and placed in a whirl-pack bag for either RNA or protein analysis, flash frozen, and placed in a cooler of dry ice. Samples were then shipped to Texas Tech University for analysis and were subsequently stored in −80 °C freezer until analysis.

### RNA Isolation and Real-Time Quantitative Reverse Transcription Polymerase Chain Reaction

Ribonucleic acid from muscle and adipose tissue was isolated with ice-cold buffer containing TRI Reagent^®^ (Sigma, St. Louis, MO). Approximately 1.5 g of frozen tissue was homogenized with TRI Reagent^®^ at a ratio of 0.5:1 grams of tissue to milliliter reagent. The homogenate was then pipetted into two microcentrifuge tubes (1 mL sample per tube), 200 μL chloroform was added to each tube, vortexed for 30 s, and incubated for 5 min The sample was then centrifuged at 15,000×*g* for 15 min separating the sample into three layers. The top supernatant layer was pipetted off and placed into new microcentrifuge tubes. Ice-cold isopropyl alcohol (250 μL) was added to the supernatant, shaken, and incubated for 10 min at 25 °C. The samples were then centrifuged at 15,000×*g* for 10 min. The supernatant was poured off, the RNA pellet at the bottom of each tube were allowed to dry, and 500 μL of 75 % ethanol was added to each tube to rinse and suspend the RNA pellet. Samples were then placed in a −80 °C freezer until needed (no longer than 3 months). Samples were then removed from the freezer and thawed on ice. Samples were then centrifuged at 15,000×RPM for 10 min, ethanol was poured off, and the pellet was air dried. Nuclease-free water (30 μL) was then added to each sample to dissolve the RNA pellet. The concentration of RNA was determined with a spectrophotometer at an absorbance of 260 nm using a NanoDrop 1000 (NanoDrop products, Wilmington, DE). Samples were then treated with DNAse to remove any DNA contaminants using a DNA-free kit (Life Technologies, Grand Island, NY). The RNA was then subjected to reverse transcription to produce cDNA. The cDNA was then used for real-time quantitative reverse transcription-PCR (RT-qPCR) to measure the abundance of AMP-activated protein kinase alpha (AMPKα) , beta 1 adrenergic receptor (β1AR), beta 2 adrenergic receptor (β2AR), and beta 3 adrenergic receptor (β3AR), myosin heavy chain (MHC)-I, MHC-IIA, MHC-IIX, C-enhancer binding protein beta (CEBPβ), G protein-coupled receptor 43 (GPR43), G protein-coupled receptor 41 (GPR41), glucose transporter type 4 (Glut4), peroxisome proliferator-activated receptor gamma (PPARγ), and stearoyl-CoA desaturase (SCD) mRNA relative to the abundance of ribosomal protein subunit 9 (RPS9) mRNA in total RNA isolated from muscle tissue. Real-time qPCR was used to measure the abundance of AMPKα, β2AR, GPR43, GPR41, Glut4, SCD, CEBPβ, and PPARγ mRNA relative to the abundance of RPS9 mRNA in total RNA isolated from adipose tissue. Bovine primers and probes for AMPKα, β1AR, β2AR, β3AR, MHC-I, MHC-IIA, MHC-IIX, GPR43, GPR41, Glut4, SCD, CEBPβ, and PPARγ are presented in Table 3. Assays were performed in the GeneAmp 7900HT Sequence Detection System (Applied Biosystems, Life Technologies) using thermal cycling parameters recommended by the manufacturer (40 cycles of 15 s at 95 °C and 1 min at 60 °C).

**Table 3:** Sequence of bovine-specific PCR primers and TaqMan probes to be used for determination of expression of mRNA of AMPKα, MHC-I, MHC-IIA, MHC-IIX, β1AR, β2AR, β3AR, CEBPβ, GPR43, GPR41, Glut4, PPARγ, SCD, and RPS9

Primer	Sequence (5′ to 3′)
AMPkα (accession # NM_001109802)	
Forward	ACCATTCTTGGTTGCTGAAACTC
Reverse	CACCTTGGTGTTTGGATTTCTG
TaqMan probe	6FAM-CAGGGCGCGCCATACCCTTG-TAMRA
MHC-I (accession no. AB059400)	
Forward	CCCACTTCTCCCTGATCCACTAC
Reverse	TTGAGCGGGTCTTTGTTTTTCT
TaqMan probe	6FAM-CCGGCACGGTGGACTACAACATCATAG-TAMRA
MHC-IIA (accession no. AB059398)	
Forward	GCAATGTGGAAACGATCTCTAAAGC
Reverse	GCTGCTGCTCCTCCTCCTG
TaqMan probe	6FAM-TCTGGAGGACCAAGTGAACGAGCTGA-TAMRA
MHC-IIX (accession no. AB059399)	
Forward	GGCCCACTTCTCCCTCATTC
Reverse	CCGACCACCGTCTCATTCA
TaqMan probe	6FAM-CGGGCACTGTGGACTACAACATTACT-TAMRA
β1AR (accession no. AF188187)	
Forward	GTGGGACCGCTGGGAGTAT
Reverse	TGACACACAGGGTCTCAATGC
TaqMan probe	6FAM-CTCCTTCTTCTGCGAGCTCTGGACCTC-TAMRA
β2AR (accession no. NM_174231)	
Forward	CAGCTCCAGAAGATCGACAAATC
Reverse	CTGCTCCACTTGACTGACGTTT
TaqMan probe	6FAM-AGGGCCGCTTCCATGCCC-TAMRA
β3AR (accession no. X85961)	
Forward	AGGCAACCTGCTGGTAATCG
Reverse	GTCACGAACACGTTGGTCATG
TaqMan probe	6FAM-CCCGGACGCCGAGACTCCAG-TAMRA
CEBPβ (accession no. NM_176788)	
Forward	CCAGAAGAAGGTGGAGCAACTG
Reverse	TCGGGCAGCGTCTTGAAC
TaqMan probe	6FAM-CGCGAGGTCAGCACCCTGC-TAMRA
GPR43 (accession no. FJ562212)	
Forward	GGCTTTCCCCGTGCAGTA
Reverse	ATCAGAGCAGCCATCACTCCAT
TaqMan probe	6FAM-AAGCTGTCCCGCCGGCCC-TAMRA
GPR41 (accession no. FJ562213)	
Forward	TGCTCCTCAGCACCCTGAA
Reverse	TTGGAACCCAGATGATGAGAAA
TaqMan probe	6FAM-TCCTGCGTCGACCCCCTTGTCTAC-TAMRA
Glut4 (accession no. D63150)	
Forward	CCTCGGCAGCGAGTCACT
Reverse	AAACTGCAGGGAGCCAAGAA
TaqMan probe	6FAM-CCTTGGTCCTTGGCGTATTCTCCGC-TAMRA
PPARγ (accession no. NM_181024)	
Forward	ATCTGCTGCAAGCCTTGGA
Reverse	TGGAGCAGCTTGGCAAAGA
TaqMan probe	6FAM-CTGAACCACCCCGAGTCCTCCCAG-TAMRA
SCD (accession no. AB075020)	
Forward	TGCCCACCACAAGTTTTCAG
Reverse	GCCAACCCACGTGAGAGAAG
TaqMan probe	6FAM-CCGACCCCCACAATTCCCG-TAMRA
RPS9 (accession no. DT860044)	
Forward	GAGCTGGGTTTGTCGCAAAA
Reverse	GGTCGAGGCGGGACTTCT
TaqMan probe	6FAM-ATGTGACCCCGCGGAGACCCTTC-TAMRA

*AMPKα* AMP-activated protein kinase alpha, *MHC-I* myosin heavy chain-I, *MHC-IIA* myosin heavy chain-IIA, *MHC-IIX* myosin heavy chain-IIX, *β1AR* beta 1 adrenergic receptor, *β2AR* beta 2 adrenergic receptor, *β3AR* beta 3 adrenergic receptor, *CEBPβ* C-enhancer binding protein beta, *GPR43* G-protein coupled receptor 43, *GPR41* G-protein coupled receptor 41, *Glut4* glucose transporter type 4, *PPARγ* peroxisome proliferator-activated receptor gamma, *SCD* stearoyl-CoA desaturase and RPS9 ribosomal protein S9

### Protein Extraction, Western Blots, and SDS-PAGE Gel Electrophoresis

Protein from muscle was isolated with whole muscle extraction buffer (WMEB; 2 % sodium dodecyl sulfate, 10 mM phosphate, pH 7.0). Adipose tissue protein was isolated with an ice-cold buffer containing tissue protein extraction reagent (T-PER; Fisher Scientific, Fair Lawn, NJ), protein inhibitor (Roche, Branchburg, NJ), and 2 mM Na_3_VO_4_ (Fisher Scientific) at a 1:5 ratio. The homogenized samples were centrifuged at 15,000×*g* for 15 min, separating the sample into three layers. The middle supernatant layer was pipetted off and placed into microcentrifuge tubes. The protein samples were then diluted with either T-PER or WMEB to determine protein concentration using the Pierce^™^ BCA^™^ protein assay (Thermo Fisher Scientific, Fairlawn, NJ). Protein concentration was then determined using a NanoDrop 1000 spectrophotometer at 562 nm. All samples were then diluted to the same concentration. Modified Wang’s tracking dye was added to western blot samples, and MHC tracking dye was added to sodium dodecyl sulfate polyacrylamide gel electrophoresis (SDS-PAGE) samples. Samples were denatured with β-mercaptoethanol and incubated for 2 min at 95 °C. Samples for western blots were then loaded onto Novex 4–12 % Bis-Tris gels (Invitrogen, Grand Island, NY), and protein was separated by gel electrophoresis. The gels were run for approximately 35 min at 165 V and 27 mA. Proteins were transferred onto a nitrocellulose membrane (Invitrogen) for 7 min. Following transfer, the membrane was incubated with non-fat dry milk (BIO RAD, Hercules, CA), 10 % 10 × tris-buffered saline (TBS) in NanoPure water for 1 h at 25 °C to block non-specific antibody binding. The blocking solution was then removed from the membrane. The appropriate primary antibodies 1:1000 α-beta 1 AR, rabbit, IgG (abcam^®^, Cambridge, MA); 1:1000 α-beta 2 AR, goat, IgG (abcam); and 1:1000 α-beta 3 AR, goat, IgG (abcam) were mixed into 1 × TBS-Tween solution, added to the membrane and allowed to incubate for 2 h (β1AR) or 1 h (β2AR and β3AR) at 25 °C. The membrane was then rinsed three times for 10 min in TBS-Tween. The appropriate Alexa fluorescent antibodies goat α-rabbit, IgG, Alexa-Fluor 633 (Invitrogen) and donkey α-goat, IgG, Alexa-Fluor 633 (Invitrogen) were then added at a dilution of 1:2000 in TBS-Tween to the membrane and incubated for 1 h at 25 °C in the absence of light. The membranes were then rinsed three times for 10 min in TBS-Tween in unlighted conditions. The membranes were then dried and visualized using the Imager Scanner II and ImageQuant TL programs. Densitometry measurements were made on the bands corresponding to β1AR, β2AR, and β3AR using a molecular weight standard for reference (Precision Plus Protein^™^ All Blue Standards; BIO RAD).

For SDS-PAGE, 6 % acrylamide separating gels with 4 % acrylamide stacking gels were made and set at 4 °C for 4–24 h. Samples were then loaded onto the gels, and protein was separated by gel electrophoresis. The gels were run for approximately 72 h at 100 V. The gel was placed in 300 mL Coomassie^®^ Fluor Orange (Life Technologies) for 30 min at 25 °C in an opaque container. The Coomassie Fluor Orange was drained off the gel, and the gel was briefly rinsed in 7.5 % acetic acid followed by NanoPure water. The gels were then visualized using the Imager Scanner II and ImageQuant TL programs. Densitometry measurements were made on the bands corresponding to MHC-II and MHC-I.

### Immunohistochemical Analysis

Twenty-four hours prior to sectioning, embedded muscle samples were moved from −80 to a −20 °C freezer to thaw. Muscle fiber distribution, area, β-adrenergic receptor, and satellite cell abundance were determined on 10-μm thick cross sections. The sections were cut at −20 °C using a Leica CM1950 cryostat (Lieca Biosystems, Buffalo Grove, IL) from the embedded muscle samples. The sections were then mounted on positively charged glass slides (five slides per sample/three cryosections per slide; Superfrost Plus; VWR International). Cryosections were fixed using 4 % paraformaldehyde (Thermo Fisher Scientific) for 10 min at 25 °C followed by two brief rinses and a single 5-min rinse in phosphate buffered saline (PBS). Cryosections were incubated with 5 % horse serum (Invitrogen), 2 % bovine serum albumin (MP Biomedical, Solon, OH), and 0.2 % Triton-X100 (Thermo Fisher Scientific) in PBS for 30 min at 25 °C to block non-specific antibody binding. Cryosections were then incubated for 1 h at 25 °C in the following primary antibodies: slide 1, 1:100 α-dystrophin, rabbit, IgG (Thermo Scientific); 1:100 supernatant anti-MHC type 1, IgG2b (BA-D5; Developmental Studies Hybridoma Bank, University of Iowa, Iowa City, IA); and supernatant anti-MHC (all but type IIX IgG1; BF-35, Developmental Studies Hybridoma Bank); slide 2, 1:750 α-beta 1 AR, rabbit, IgG (abcam); 1:750 α-beta 2 AR, chicken, IgY (abcam); 1:500 α-beta 3 AR, goat, IgG (abcam); slide 3, 1:10 supernatant anti-paired box protein 7 (Pax7), mouse α-chicken (Developmental Studies Hybridoma Bank); 1:100 myogenic factor 5 (Myf-5), rabbit, IgG (Santa Cruz Biotechnology, Dallas, TX). Slides were then rinsed three times for 5 min in PBS. Cryosections were incubated for 30 min at 25 °C in opaque boxes in the following secondary antibodies: slide 1, 1:1000 goat α-rabbit, IgG, Alexa-Fluor 488 (Invitrogen); 1:1000 goat α-mouse, IgG1, Alexa-Fluor 546 (Invitrogen); 1:1000 goat α-mouse, IgG2b, Alexa-Fluor 633 (Invitrogen); slide 2, 1:1000 goat α-chicken, IgY, H & L, Alexa-Fluor 488 (abcam); 1:1000 donkey α-rabbit, IgG, Alexa-Fluor 546 (Invitrogen); 1:1 000 donkey α-goat, IgG, Alexa-Fluor 633 (Invitrogen); slide 3, 1:1000 goat α-rabbit, IgG, Alexa-Fluor 488 (Invitrogen); 1:1000 goat α-mouse, IgG1, Alexa-Fluor 546 (Invitrogen). Slides were then rinsed three times for 5 min in PBS. Finally, cryosections were incubated in 1 μg/mL 4′,6-diamidino-2-phenylindole (DAPI, Thermo Fisher Scientific) for 1 min followed by two brief PBS rinses. Slides were cover-slipped with mounting media (Aqua Mount; Lerner Laboratories, Pittsburgh, PA) and thin glass cover slips (VWR International), and dried at 4 °C for 24 h. All slides were imaged within 48 h of staining.

The slides were imaged at ×200 working difference magnification using an inverted fluorescence microscope (Nikon Eclipse, Ti-E; Nikon Instruments Inc., Mellville, NY) equipped with a UV light source (Nikon Intensilight Inc.; C-HGFIE). The images were captured by a CoolSnap ES^2^ monochrome camera and artificially colored and analyzed using the NIS Elements^®^ Imaging software.

Five random images were taken of cryosections from each slide of the *semimembranosus* tissue. All MHC type I, IIA, and IIX muscle fibers in each image were identified and expressed as a percentage of the total number of muscle fibers. The cross-sectional area of each fiber in each image was measured using the NIS Elements software (Nikon Instruments Inc.) and expressed on a square millimeter basis. The total number of DAPI-stained cells in each image were enumerated to determine the nuclear density on a per square millimeter basis. All βAR, Pax7, Myf5, and, Pax7 + Myf5 satellite cells were identified on the respective slides stained for them, counted, and densities are reported on a square mm basis. Beta-adrenergic receptors were classified as βAR or internalized βAR. Classification was determined by the location of the stained receptors on the fiber cross-section. Receptors located on sarcolemma were considered normal βAR, and the receptors located within the fiber cross-section were considered internalized βAR.

### Fatty Acid Analysis

Fatty acids (FA) were determined for the polar (PL) and neutral lipids (NL) of each muscle tissue sample [9]. Muscle tissue was cubed, flash-frozen in liquid nitrogen, and homogenized into fine powder. All tissue homogenates were stored at −80 °C until subsequent analysis. Total lipids were extracted from 0.5-g tissue homogenates by a chloroform:methanol extraction [2]. Extracted lipids were fractionated using a Resprep^®^ silica gel cartridge (Restek Corporation, Bellefonte, PA), where NL were initially eluted with chloroform, and PL were subsequently eluted with methanol [7]. Fatty acids of the NL were saponified and derivatized to fatty acid methyl esters (FAME) using sodium methoxide in methanol [10]. Saponification and derivatization of PL FA was carried out with methanolic potassium hydroxide [11]. Tridecanoic acid methyl ester (CAS# 1731-88-0, Sigma-Aldrich) was used as the internal standard during derivatization. Analysis of FAME was carried out by an Agilent Technologies (Santa Clara, CA) 7890 gas chromatograph equipped with an HP-88 capillary column (30 m × 250 μm × 0.2 μm; Agilent Technologies, Santa Clara, CA) and a flame ionization detector. Identity of FAME was determined by comparison with authentic FAME standards (Supelco^®^ 37 Component FAME Mix, Sigma-Aldrich, St Louis, MO) and quantified by an internal standard calibration. Individual FA were calculated as milligram per gram of muscle tissue. FA were added to calculate the total FA concentration (mg/g muscle tissue) of each fraction and the entire FA composition. Percentages of FA were determined by dividing the individual FA concentration (mg/g muscle tissue) by the corresponding total FA concentration (mg/g muscle tissue) then multiplying by 100. Percentages of NL and PL fractions were calculated by dividing the lipid fraction concentrations (mg/g muscle tissue) by the total FA concentration (mg/g muscle tissue).

### Statistical Analysis

Performance and carcass data were analyzed using the GLIMMIX procedure of SAS (v.9.3, SAS Institute; Carey, NC). The model included block as a random effect, and treatment served as a fixed effect. Pen served as the experimental unit for feedlot performance and carcass characteristics. Initial weight was used as a covariate. Treatment means were separated using the LSMEANS procedure with PDIFF option and considered different at *P* < 0.05. Tendencies for differences among treatment means were declared when 0.05 > *P* ≤ 0.10. Yield grade and quality grade distributions were analyzed using the FREQUENCY procedure of SAS using the chi-square option.

For all biochemical analysis, data were analyzed using the GLIMMIX procedure of SAS (v.9.3, SAS Institute; Carey, NC). The model included treatment as the fixed effect, steer served as the experimental unit, and the Kenward-Roger adjustment was used to correct degrees of freedom. Means were separated using the LSMEANS procedure with the PDIFF option and considered different when *P* ≤ 0.05. Tendencies for differences among treatment means were declared when 0.05 > *P* ≤ 0.10.

## Results and Discussion

There were no differences (*P* > 0.05; Table 4) in starting and final weights, dry matter intake (DMI), average daily gain (ADG), and feed to gain ratios. Spears [24] reported differences in weight of heifers supplemented with ZnO or ZnMet when compared to control. However, when ZnO and ZnMet were supplemented to lambs, ADG and F:G were improved with Zn supplementation [24]. Zinc methionine has been reported to increase rate of gain and feed efficiency of heifers [25]. Greene [3] and Rust [21] reported no difference in gain or feed efficiency when steers were supplemented ZnO or ZnMet. In a more recent study, the addition of Zn on ADG in heifers was unaffected [6]. However, there was an interaction between Zn and implant when Zn was supplemented to heifers and steers [6]. In the heifers, ADG was 26 % greater in non-implanted heifers fed with ZnMet than implanted heifers fed with ZnMet [6]. When ZnSO_4_ was the supplemented Zn source, it did not have an effect on ADG, and the implanted heifers had a greater ADG regardless of ZnSO_4_ supplementation [6]. When steers were implanted and supplemented a Zn source, the results were different [6]. Implant improved ADG in steers fed with a control diet or supplemented with ZnSO_4_, but did not affect ADG of implanted steers supplemented with ZnMet [6]. Furthermore, in the current study, there was no difference (*P* > 0.05; Table 5) in HCW, DP, LMA, MS, and YG between the ZnMet and CON cattle. There were no differences between YG and QG distributions (*P* > 0.05; Table 6). Greene et al. [3] reported that ZnMet increased the percent of kidney, pelvic and heart fat, MS, and overall QG and tended to increase backfat thickness. Rust [21] reported that steers supplemented with ZnMet graded 47 % USDA Choice compared to control at 37 %, and Greene et al. [3] reported ZnMet steers graded 79 % USDA Choice compared to control at 57 % and ZnO at 40 %. While our distributions were not significant (*P* = 0.134), 4 % of ZnMet supplemented steers graded USDA Prime compared to the 1 % of CON. Huerta et al. [6] reported that heifers supplemented ZnMet graded 70 % USDA Choice compared to 50 % USDA Choice in control heifers and 30 % USDA Choice in ZnSO_4_ heifers. However, there were no differences in carcass grades or other carcass characteristics of steers supplemented with ZnMet or ZnSO_4_ [6]. To our knowledge, no other published literature has evaluated the effects of ZnMet supplementation and ZH feeding in beef cattle.

**Table 4:** Effect of zinc methionine complex in combination with zilpaterol hydrochloride on growth performance of calf-fed Holstein steers

	CON^a^	ZnMet^b^	SEM^c^	*P* value
Start (wt, kg)	482	481	3.54	0.837
Final (wt, kg)	618	615	4.81	0.634
Pen start (wt, kg)	52057	51974	382.63	0.838
Pen final (wt, kg)	65539	65425	1041.42	0.916
Mortality, *n*	3	1	0.88	0.309
DOF	114	114	0.45	0.602
DMI (kg)	8.38	8.40	0.04	0.666
ADG (kg)	1.14	1.16	0.04	0.750
F:G	7.61	7.34	0.33	0.465
G:F	0.13	0.13	0.01	0.638

^a^CON: Control 90 ppm Zn added from ZnSO_4_ (*n* = 6 pens).

^b^ZnMet: Control plus 720 mg Zn added from ZINPRO^®^ zinc methionine (*n* = 6 pens).

^c^Pooled standard error of the mean

**Table 5:** Effect of zinc methionine complex in combination with zilpaterol hydrochloride on carcass characteristics of calf-fed Holstein steers

	CON^a^	ZnMet^b^	SEM^d^	*P* value
HCW (kg)	389	388	2.75	0.794
Dress (%)	62.9	63.0	0.53	0.858
Marbling^c^	475	487	6.83	0.139
LMA (cm^2^)	82.35	81.83	0.47	0.317
USDA yield grade	2.8	2.8	0.03	0.835

^a^CON: Control 90 ppm Zn added from ZnSO_4_ (*n* = 6 pens)

^b^ZnMet: Control plus 720 mg Zn added from ZINPRO^®^ zinc methionine (*n* = 6 pens)

^c^Marbling score 300 = slight; 400 = small; 500 = modest

^d^Pooled standard error of the mean

**Table 6:** Effect of zinc methionine complex in combination with zilpaterol hydrochloride on USDA quality and yield grade of calf-fed Holstein steers

Grade, % of carcasses	CON^a^	ZnMet^b^	SEM^c^	*P* value
Prime	1.30	4.35	1.70	0.134
Choice	78.39	76.07	2.66	0.423
Select	19.78	18.75	2.38	0.682
No roll	0.52	0.82	0.32	0.396
Yield grade 1	6.13	3.96	1.97	0.322
Yield grade 2	57.90	62.40	2.74	0.161
Yield grade 3	34.96	33.13	3.70	0.642
Yield grade 4	1.20	1.20	0.48	1.000

^a^CON: Control 90 ppm Zn added from ZnSO_4_ (*n* = 6 pens)

^b^ZnMet: Control plus 720 mg Zn added from ZINPRO^®^ zinc methionine (*n* = 6 pens)

^c^Pooled standard error of the mean

In the *semimembranosus* tissue, ZnMet cattle contained a greater abundance of MHC-I mRNA (*P* < 0.05; Table 7) and tended to have a greater abundance of β2AR mRNA (*P* < 0.10; Table 7). The ZnMet-supplemented cattle had a lower (*P* < 0.05) abundance of MHC-IIX and β1AR mRNA. Peroxisome proliferator-activated receptor gamma and SCD mRNA abundance were greater (*P* < 0.05) in CON cattle. There were no changes (*P* > 0.05; Table 7) in AMPKα, MHC-IIA, β2AR, β3AR, CEBPβ, GPR43, GPR41, and Glut4 mRNA between treatments. Knobel [8] reported that ZH increased MHC-IIX in the *gluteus medius* supporting the results seen in our CON group. In data collected from our laboratory in bovine satellite cells treated with 0 μM ZH and 0 μM Zn, 10 μM ZH, 1 μM Zn, or 10 μM ZH and 1 μM Zn, we discovered that the combination of Zn and ZH 96 h post-treatment decreased MHC-IIX and tended to increase MHC-I mRNA abundance [5] reflecting the results of this trial. In bovine satellite cell culture work, the addition of ractopamine HCl (RH) and Zn had no effect on β1AR, β2AR, AMPKα, IGF-1, MHC-I, MHC-IIA, and MHC-IIX mRNA abundance [4]. Ractopamine HCl is another βAA used in beef and pork production. Analysis of the adipose tissue in this study revealed a greater abundance of Glut4 mRNA in CON cattle (*P* < 0.05; Table 8) when compared to ZnMet. Analysis of AMPKα, β2AR, CEBPβ, GPR43, GPR41, PPARγ, and SCD revealed no differences (*P* > 0.05) between treatments. Oh and Choi [15] reported an increase in PPAYγ2 mRNA expression in bovine intramuscular adipocytes when Zn sources were added to the differentiation media at a concentration of 50 and 100 μM.

**Table 7:** Effect of zinc methionine complex in combination with zilpaterol hydrochloride on relative mRNA concentrations of AMPKα, MHC-I, MHC-IIA, MHC-IIX, β1AR, β2AR, β3AR, CEBPβ, GPR43, GPR41, Glut4, PPARγ, and SCD genes in *semimembranosus* tissue

Gene^a^	CON^b^	ZnMet^c^	SEM^d^	*P* value
AMPkα	1.746	1.613	0.207	0.525
MHC-I	0.649	1.025	0.166	0.030
MHC-IIA	2.517	2.208	0.438	0.485
MHC-IIX	1.514	1.085	0.165	0.013
β1AR	7.163	3.101	1.457	0.008
β2AR	0.894	1.282	0.194	0.053
β3AR	33.133	41.718	9.752	0.397
CEBPβ	5.670	4.838	1.157	0.476
GPR43	2.391	0.873	1.292	0.247
GPR41	38.694	38.257	16.807	0.979
Glut4	1.035	1.123	0.095	0.363
PPARγ	3.020	1.749	0.540	0.024
SCD	9.745	2.303	3.052	0.019

*AMPKα* AMP-activated protein kinase alpha, *MHC-I* myosin heavy chain-I, *MHC-IIA* myosin heavy chain-IIA, *MHC-IIX* myosin heavy chain-IIX, *β1AR* beta 1 adrenergic receptor, *β2AR* beta 2 adrenergic receptor, *β3AR* beta 3 adrenergic receptor, *CEBPβ* C-enhancer binding protein beta, *GPR43* G-protein coupled receptor 43, *GPR41* G-protein coupled receptor 41, *Glut4* glucose transporter type 4, *PPARγ* peroxisome proliferator-activated receptor gamma, *SCD* stearoyl-CoA desaturase

^a^Relative abundance of the AMPKα, MHC-I, MHC-IIA, MHC-IIX, β1AR, β2AR, β3AR, CEBPβ, GPR43, GPR41, Glut4, PPARγ, and SCD genes were normalized with the RPS9 endogenous control by using the change in cycle threshold (ΔCT)

^b^CON: Control 90 ppm Zn added from ZnSO_4_ (*n* = 20 steers)

^c^ZnMet: Control plus 720 mg Zn added from ZINPRO^®^ zinc methionine (*n* = 20 steers)

^d^Pooled standard error of the mean

**Table 8:** Effect of zinc methionine complex in combination with zilpaterol hydrochloride on relative mRNA concentrations of AMPKα, β2AR, CEBPβ, GPR43, Glut4, PPARγ, and SCD genes in subcutaneous adipose tissue

Gene^a^	CON^b^	ZnMet^c^	SEM^d^	*P* value
AMPkα	0.944	0.895	0.130	0.708
β2AR	0.947	0.866	0.263	0.758
CEBPβ	0.467	0.675	0.231	0.373
GPR43	210.230	356.230	169.240	0.400
Glut4	0.843	0.545	0.136	0.035
PPARγ	0.895	0.999	0.176	0.558
SCD	2.358	2.355	1.016	0.997

*AMPKα* AMP-activated protein kinase alpha, *β2AR* beta 2 adrenergic receptor, *CEBPβ* C-enhancer binding protein beta, *GPR43* G-protein coupled receptor 43, *GPR41* G-protein coupled receptor 41, *Glut4* glucose transporter type 4, *PPARγ* peroxisome proliferator-activated receptor gamma, *SCD* stearoyl-CoA desaturase

^a^Relative abundance of the AMPKα, β2AR, CEBPβ, GPR43, GPR41, Glut4, PPARγ, and SCD genes were normalized with the RPS9 endogenous control by using the change in cycle threshold (ΔCT)

^b^CON: Control 90 ppm Zn added from ZnSO_4_ (*n* = 20 steers)

^c^ZnMet: Control plus 720 mg Zn added from ZINPRO^®^ zinc methionine (*n* = 20 steers)

^d^Pooled standard error of the mean

**Fig. 1:**
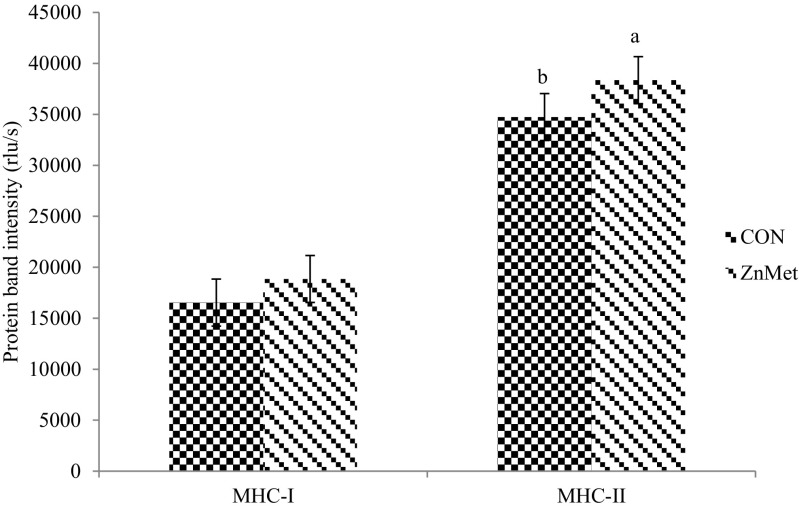
Effect of zinc methionine complex in combination with zilpaterol hydrochloride on relative protein concentration of myosin heavy chain (MHC)-I and II *semimembranosus* tissue. CON: Control 90 ppm Zn added from ZnSO_4_ (*n* = 20 steers). ZnMet: Control plus 720 mg Zn added from ZINPRO^®^ zinc methionine (*n* = 20 steers). There was no difference in protein concentration of MHC-I (*P* = 0.322; pooled standard error of the mean (SEM) = 2305.27). There was a significant difference in the protein concentration of MHC-II (*P* = 0.001; SEM = 2021.73)

**Fig. 2:**
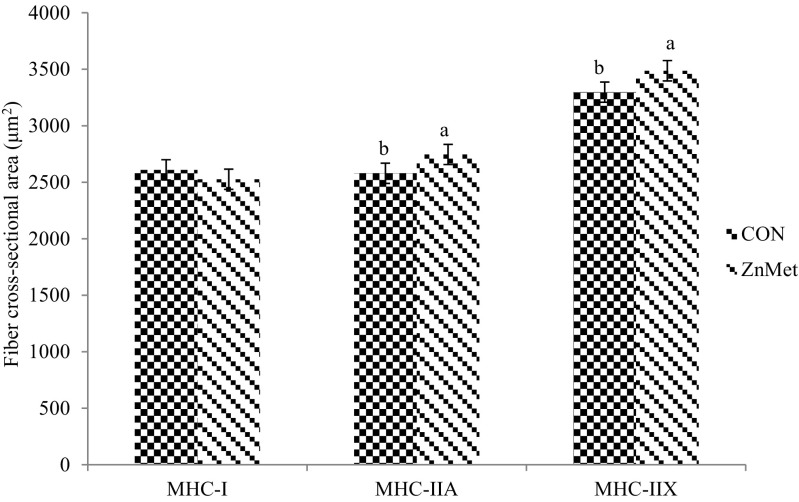
Effect of zinc methionine complex in combination with zilpaterol hydrochloride on fiber cross-sectional area, (μm^2^) in *semimembranosus* tissue. CON: Control 90 ppm Zn added from ZnSO_4_ (*n* = 20 steers). ZnMet: Control plus 720 mg Zn added from ZINPRO^®^ zinc methionine (*n* = 20 steers). There was no difference in fiber cross-sectional area of MHC-I (*P* = 0.353; pooled standard error of the mean (SEM) = 89.756). There was a significant difference in the fiber cross-sectional area of MHC-IIA (*P* = 0.001; SEM = 50.977) and MHC-IIX (*P* = 0.001; SEM = 59.431)

No differences were detected (*P* > 0.05; Table 9) in β1AR, β2AR, and β3AR protein abundance between treatments in the *semimembranosus* tissue. There were also no differences (*P* > 0.05; Table 9) in β2AR and β3AR protein abundance between treatments in adipose tissue. These results are supported by in vitro data from our laboratory, where we found no difference in β1AR or β2AR protein abundance of cells treated with ZH and Zn [3]. Furthermore, Harris [4] also reported no changes in β1AR and β2AR protein abundance of cells treated with RH and Zn. Zinc methionine cattle had greater (*P* < 0.05; Fig. 1) MHC-II protein abundance than CON. The MHC-II protein abundance includes type IIA and IIX. In addition, ZnMet cattle had an increased fiber cross-sectional area of MHC-IIA and IIX fibers (*P* < 0.05; Fig. 2). There was no difference (*P* > 0.05; Fig. 1) in MHC-I protein abundance and fiber cross-sectional area (*P* > 0.05; Fig. 2) between treatments.

**Table 9:** Effect of zinc methionine complex in combination with zilpaterol hydrochloride on relative protein concentration of β1AR, β2AR, and β3AR in *semimembranosus* and adipose tissue

Receptor	CON^a^	ZnMet^b^	SEM^c^	*P* value
*Semimembranosus*				
β_1_	3435	3311	109.34	0.266
β_2_	31,924	32,008	1018.98	0.934
β_3_	9235	9612	461.14	0.418
Adipose				
β_2_	36,354	36,198	1429.45	0.913
β_3_	35,159	34,662	1027.68	0.631

^a^CON: Control 90 ppm Zn added from ZnSO_4_ (*n* = 20 steers)

^b^ZnMet: Control plus 720 mg Zn added from ZINPRO^®^ zinc methionine (*n* = 20 steers)

^c^Pooled standard error of the mean

**Fig. 3:**
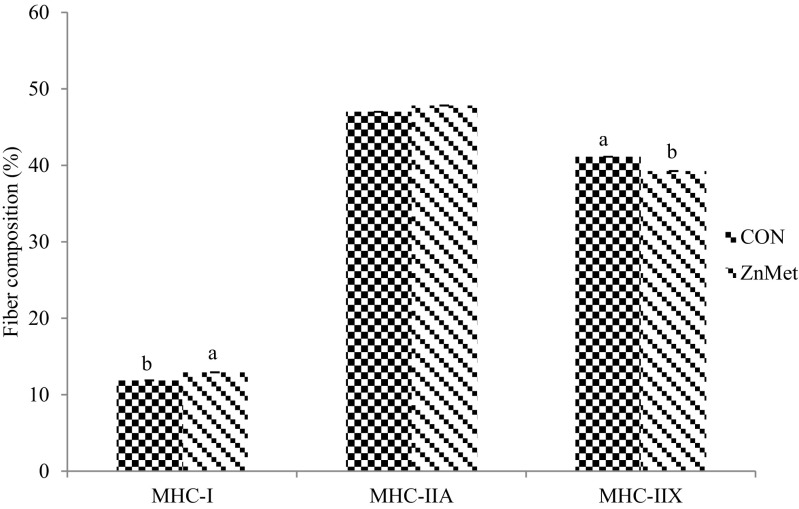
Effect of zinc methionine complex in combination with zilpaterol hydrochloride on muscle fiber type composition in *semimembranosus* tissue. CON Control 90 ppm Zn added from ZnSO_4_ (*n* = 20 steers). ZnMet Control plus 720 mg Zn added from ZINPRO^®^ zinc methionine (*n* = 20 steers). There was a significant difference in composition of MHC-I (*P* = 0.001; pooled standard error of the mean (SEM) = 0.026) and MHC-IIX (*P* = 0.001; SEM = 0.017). There was no difference in the composition of MHC-IIA (*P* = 0.069; SEM = 0.017)

Likewise, ZnMet cattle had a greater percentage of MHC-I fibers (*P* < 0.05; Fig. 3), and tended to have a greater percentage of MHC-IIA fibers (*P* < 0.10; 47.01 vs 47.83 % respectively). Control cattle had a greater percentage of MHC-IIX fibers (*P* < 0.05; Fig. 3). Zilpaterol HCl has been shown to increase the percentage of MHC-IIX fibers in the *longissimus lumborum*, increase the percentage of MHC-IIX, and decrease the percentage of MHC-I in the *gluteus medius* [8]. Paulk et al. [16] reported a linear decrease in the percent of MHC-IIA fibers as Zn concentration increased in pigs supplemented with RH and Zn. There was a tendency for the percentage of MHC-IIX fibers to increase when supplemental Zn was fed in combination with RH [16].

Cattle not supplemented with ZnMet had the greater density of nuclei (*P* < 0.05; Table 10) and ZnMet cattle had a greater density of cells expressing Pax7 (*P* < 0.005; Table 10). There was no difference in the density of cells expressing Myf5 and Pax7/Myf5 between treatments (*P* > 0.05). Knobel [8] reported that ZH supplementation decreased nuclei density in the *longissimus lumborum*, *gluteus medius*, and *semimembranosus*. Zilpaterol HCl did not affect satellite cell populations in the *longissimus lumborum*, *gluteus medius*, and *semimembranosus* [8].

**Table 10:** Effect of zinc methionine complex in combination with zilpaterol hydrochloride on nuclei and satellite cell density in *semimembranosus* tissue

Item (mm^2^)	CON^a^	ZnMet^b^	SEM^c^	*P* value
Total nuclei	658.99	596.10	16.902	0.001
Myofiber nuclei	577.58	509.38	16.806	<0.001
Pax7	1.81	3.71	0.669	0.005
Myf5	66.66	70.84	3.245	0.199
Pax7 + Myf5	12.92	12.16	1.583	0.628

^a^CON: Control 90 ppm Zn added from ZnSO_4_ (*n* = 20 steers)

^b^ZnMet: Control plus 720 mg Zn added from ZINPRO^®^ zinc methionine (*n* = 20 steers)

^c^Pooled standard error of the mean

When evaluating β1AR, internalized β1AR and internalized β3AR, no differences were detected (*P* > 0.05; Table 11). Control cattle had a greater density of β2AR (*P* < 0.05), while ZnMet cattle tended to have a greater density of internalized β2AR (*P* < 0.10). Furthermore, ZnMet cattle tended to have a greater density of β3AR (*P* < 0.10). These data indicate combining ZnMet and ZH did not affect growth performance; however, the combination of these supplements numerically increased the marbling score and the percentage of cattle that graded USDA Prime (1.30 and 4.35 %; CON vs ZnMet, respectively). Furthermore, fatty acid concentrations from the neutral lipid fraction, primarily triglycerides from adipocytes, were determined to be greater in ZnMet muscle tissue (*P* < 0.05; Table 12). The slight increases observed in marbling score and cattle grading USDA Prime, along with increased concentrations of neutral lipid fatty acids in ZnMet cattle may be the result of decreased mRNA expression of Glut4 in subcutaneous adipose tissue. The effect of decreased mRNA expression of SCD in ZnMet cattle affected fatty acid composition and saturation. Through tabulation of fatty acids as percentages (g/100 g total fatty acids), it was determined that neutral lipid stearic acid (18:0) was greater in ZnMet (*P* < 0.05; Tables 12 and 13). Meanwhile, oleic acid (18:1) was increased in CON neutral lipids (*P* < 0.05). These results are in agreement with SCD expression (Table 7) and show a direct impact on desaturation of 18:0 to 18:1.

**Table 11:** Effect of zinc methionine complex in combination with zilpaterol hydrochloride on β-adrenergic receptor density in *semimembranosus* tissue

Item (mm^2^)	CON^a^	ZnMet^b^	SEM^c^	*P* value
β_1_AR	270.84	265.72	9.052	0.572
β_1_AR-internalized	0.13	0.26	0.189	0.476
β_2_AR	350.77	308.81	10.537	0.001
β_2_AR-internalized	0.06	0.61	0.297	0.068
β_3_AR	120.67	132.66	6.309	0.058
β_3_AR-internalized	0.06	0.26	0.148	0.175

^a^CON: Control 90 ppm Zn added from ZnSO_4_ (*n* = 20 steers)

^b^ZnMet: Control plus 720 mg Zn added from ZINPRO^®^ zinc methionine (*n* = 20 steers)

^c^Pooled standard error of the mean

**Table 12:** Effect of zinc methionine complex in combination with zilpaterol hydrochloride on concentrations (mg/g muscle tissue) and percentages (g/100 g total fatty acids) of individual neutral lipid fatty acids (FA) and FA categories (saturated fatty acids (SFA), monounsaturated (MUFA), and polyunsaturated fatty acids (PUFA)) of neutral and polar lipid fractions (LF) from *semimembranosus* tissue

FA	mg/g muscle tissue				%, g/100 g total FA			
	CON^a^	ZnMet^b^	SEM^c^	*P* value	CON^a^	ZnMet^b^	SEM^c^	*P* value
*Total FA*	22.084b	34.047a	4.907	0.046				
*SFA*	10.225	16.537	2.285	0.054	45.988b	49.375a	0.945	0.013
14:0	0.832b	1.402a	0.194	0.041	3.745	4.222	0.182	0.061
15:0	0.125	0.198	0.026	0.051	0.589	0.600	0.026	0.766
16:0	5.813	9.333	1.255	0.051	26.130b	28.051a	0.694	0.049
17:0	0.252	0.419	0.062	0.061	1.131	1.213	0.062	0.332
18:0	3.141	5.111	0.759	0.069	14.085b	15.058a	0.539	0.190
23:0	0.013a	0.008b	0.001	0.003	0.076	0.027	0.021	0.054
*MUFA*	11.044	16.208	2.500	0.142	50.128a	46.635b	0.941	0.010
14:1	0.293	0.464	0.065	0.064	1.304	1.450	0.107	0.320
16:1	0.828	1.253	0.163	0.068	3.721	3.943	0.242	0.499
17:1	0.233	0.364	0.052	0.076	1.049	1.107	0.063	0.501
18:1 *trans*	1.089b	1.929a	0.269	0.031	5.139	5.545	0.366	0.414
18:1 *cis*-9	8.469	11.976	1.972	0.204	38.302a	33.911b	1.154	0.009
20:1	0.131b	0.220a	0.027	0.026	0.611	0.678	0.030	0.115
*PUFA*	0.815b	1.302a	0.161	0.037	3.881	3.992	0.188	0.665
18:2 n-6	0.622b	0.998a	0.123	0.033	2.987	3.058	0.155	0.733
18:3 n-3	0.038b	0.066a	0.008	0.024	0.181	0.200	0.009	0.163
20:3 n-6	0.018	0.028	0.004	0.095	0.085	0.084	0.008	0.966
20:4 n-6	0.041	0.044	0.004	0.605	0.227	0.164	0.035	0.188

Means in the same row having different letters are significant at *P* ≤ 0.05 due to ZnMet/CON × LF interaction

^a^CON: Control 90 ppm Zn added from ZnSO_4_ (*n* = 20 steers)

^b^ZnMet: Control plus 720 mg Zn added from ZINPRO^®^ zinc methionine (*n* = 20 steers)

^c^Pooled standard error of the mean

**Table 13:** Effect of zinc methionine complex in combination with zilpaterol hydrochloride on concentrations (mg/g muscle tissue) and percentages (g/100 g total fatty acids) of individual polar lipid fatty acids (FA) and FA categories (saturated fatty acids (SFA), monounsaturated (MUFA), and polyunsaturated fatty acids (PUFA)) of neutral and polar lipid fractions (LF) from *semimembranosus* tissue

FA	mg/g muscle tissue				%, g/100 g total FA			
	CON^a^	ZnMet^b^	SEM^c^	*P* value	CON^a^	ZnMet^b^	SEM^c^	*P* value
*Total FA*	1.913	2.212	0.197	0.292				
*SFA*	0.752	0.909	0.084	0.197	41.523	40.829	1.207	0.678
14:0	0.041	0.051	0.007	0.318	2.271	2.278	0.206	0.980
15:0	0.016	0.017	0.001	0.280	0.791	0.821	0.043	0.619
16:0	0.413	0.482	0.053	0.369	21.421	21.393	0.577	0.972
17:0	0.011	0.013	0.002	0.678	0.630	0.568	0.065	0.498
18:0	0.280	0.336	0.028	0.172	15.873	15.163	0.826	0.536
23:0	0.014	0.011	0.001	0.255	0.758	0.691	0.137	0.727
*MUFA*	0.576	0.544	0.057	0.701	32.358	32.791	2.079	0.880
14:1	0.020	0.023	0.002	0.361	1.119	1.186	0.074	0.513
16:1	0.039	0.047	0.007	0.431	2.190	2.074	0.172	0.625
17:1	0.015	0.018	0.002	0.427	0.865	0.790	0.049	0.272
18:1 *trans*	0.055	0.071	0.009	0.194	3.038	3.282	0.248	0.480
18:1 *cis*-9	0.429	0.429	0.047	0.997	24.198	24.644	1.662	0.846
20:1	0.019	0.020	0.001	0.625	1.058	1.064	0.163	0.979
*PUFA*	0.525	0.567	0.040	0.467	26.119	26.378	2.333	0.936
18:2 n-6	0.287	0.322	0.023	0.304	14.366	14.961	1.267	0.734
18:3 n-3	0.008b	0.009a	<0.001	0.014	0.373	0.395	0.033	0.630
20:3 n-6	0.040	0.043	0.003	0.581	2.008	1.994	0.204	0.958
20:4 n-6	0.191	0.195	0.014	0.836	9.426	9.146	0.895	0.821

Means in the same row having different letters are significant at *P* ≤ 0.05 due to ZnMet/CON × LF interaction

^a^CON: Control 90 ppm Zn added from ZnSO_4_ (*n* = 20 steers)

^b^ZnMet: Control plus 720 mg Zn added from ZINPRO^®^ zinc methionine (*n* = 20 steers)

^c^Pooled standard error of the mean

While the mechanisms by which Zn enhances muscle hypertrophy when supplemented with βAA have not been fully elucidated, there is speculation that β2AR potentially have multiple allosteric binding sites for Zn [28], allowing ZH to bind more efficiently to β2AR and elicit a greater effect on muscle hypertrophy. However, overstimulation of the βAR by βAA results in receptor desensitization [31, 33]. Receptor desensitization prompts downregulation of adenylate cyclase catalytic activity, resulting in a reduction of cAMP synthesis and protein kinase A activation [32]. When the βAR become desensitized, they are sequestered within an intracellular vesicle and lose the ability to propagate the signal transduction pathway [31, 33]. The decreased density of β2AR on the cell surface and increased density of internalized β2AR in the ZnMet cattle suggests that the β2AR were desensitized and sequestered within an intracellular vesicle. The desensitization of the β2AR may have occurred due to the allosteric binding of Zn to the β2AR, increasing the affinity for ZH to bind to the receptor. While the affinity of the β2AR may have been enhanced, the hypothesized positive implications on hypertrophy and performance were not observed in this study. Thus, future research should focus on the relationship in which zinc alters affinity and its interaction with muscle hypertrophy.

When evaluating the protein abundance of MHC and cross-sectional area of the fibers, the ZnMet cattle had a greater abundance of MHC-II protein and had greater cross-sectional areas for MHC-IIA and IIX fibers. The ZnMet treatment also increased the percentage of MHC-I and IIA fibers while decreasing the percentage of MHC-IIX fibers, indicating that Zn supplementation may alter fiber type by increasing the percentage of the more oxidative fibers. The mRNA expression supports the previous statement, as the ZnMet treatment had increased expression of MHC-I and decreased expression of MHC-IIX. The increase in MHC-II protein concentration and MHC-IIA and IIX fiber cross-sectional areas observed coupled by a decreased percentage of MHC-IIX fibers in the ZnMet treatment is an interesting phenomenon. Typically, as fiber cross-sectional area increases, the fiber types will transition to the more glycolytic fiber types [17, 18]. The ZnMet treatment had a lower density of total and myofiber nuclei, which was an expected dilution effect with increases in fiber cross-sectional area. Essentially, the ZnMet treatment had a greater cross-sectional muscle fiber area being regulated by fewer nuclei. However, it is important to note that the total nuclei density reported in this study includes nuclei associated with the satellite cells. While the ZnMet cattle had the least total nuclei density, they had the greatest density of Pax7-expressing satellite cells. The expression of Pax7 is required for adult muscle satellite cells to form and proliferate [20, 22], and Pax7 satellite cells still maintain the ability to proliferate [30]. After proliferation, satellite cells either downregulate Pax7 and enter into differentiation or maintain Pax7 and stop expressing myogenic determination factor 1 and revert to a quiescent state [30]. The increased density of Pax7-expressing satellite cells in the ZnMet treatment suggests that those animals have the ability to increase the total nuclei and myofiber nuclei through the proliferation and differentiation of the Pax7 satellite cells. The ability to increase the myofiber nuclei implies possible enhancement in muscle hypertrophy in the ZnMet treatment.

While there is little data on the interactions of Zn source, βAA, and βAR on growth and the biological process involved in these changes, these compounds have been individually studied extensively. Previous data reports that ZnMet supplementation often times increases fat thickness, as well as MS and QG which may possibly have an economic impact [3, 6, 21]. However, ZnMet supplementation has been shown to have varying effects on feedlot performance. Studies have shown that ZnMet improved ADG and feed efficiency [24, 25], while others have reported no difference in ADG or feed efficiency [3, 6, 21]. Conversely, ZH supplementation has consistently been shown to improve feedlot and carcass performance [19, 26, 29, 34]. Studies have reported ZH improved feed to gain, HCW, dressing percentage, and LMA while decreasing backfat thickness [19, 26, 29, 34]. While the combination of ZnMet and ZH had positive effects on the molecular level (MHC isoforms and cross-sectional area), there were minimal effects on overall feedlot and carcass performance. The lack of feedlot and carcass performance in this study may implicate that the animal was already at its maximum potential due to ZH supplementation, and may not be capable of additional growth performance. The mechanisms by which ZnMet and ZH interact to elicit effects on performance, lipid metabolism, and myogenic activity is still unknown, and future research should be conducted to further elucidate the molecular mechanisms that impact muscle and adipose metabolism in biological processes involving ZnMet and other growth promotant technologies.
